# Deliberate Practice as a Theoretical Framework for Interprofessional Experiential Education

**DOI:** 10.3389/fphar.2016.00188

**Published:** 2016-07-07

**Authors:** Joyce M. Wang, Joseph A. Zorek

**Affiliations:** Pharmacy Practice Division, University of Wisconsin-Madison School of PharmacyMadison, WI, USA

**Keywords:** deliberate practice, interprofessional education, interprofessional collaboration, interprofessional relations, clinical education

## Abstract

**Objective:** The theory of deliberate practice has been applied to many skill-based performance activities. The primary aim of this project was to integrate synergistic principles from deliberate practice and consensus-derived competencies for interprofessional education into a framework upon which educational models to advance interprofessional experiential education (IEE) might be built.

**Methods:** CINAHL, ERIC, and MEDLINE databases were searched using the keywords “deliberate practice” and “interprofessional education,” both individually and in combination. Relevant articles were selected from the catalog based on support for the premise of the project. Defining characteristics of deliberate practice were distilled with particular emphasis on their application to the Interprofessional Education Collaborative's (IPEC) core competencies. Recommendations for IEE development were identified through the synthesis of deliberate practice principles and IPEC competencies.

**Results:** There is a high degree of synergy between deliberate practice principles and IPEC competencies. Our synthesis of the literature yielded a cyclical four-step process to advance IEE: (1) implement an IEE plan guided by the student's strengths/weaknesses and in consideration of the collaborative practice skills they wish to develop, (2) engage in IPE experiences that will challenge targeted skills according to the IEE plan, (3) embed frequent opportunities for student reflection and preceptor/team feedback within IEE plan, and (4) revise the IEE plan and the IPE experience based on insights gained during step 3.

**Conclusion:** The cyclical four-step process synthesized through this literature review may be used to guide the development of new IEE models. The purposeful development of IEE models grounded in a theory that has already been operationalized in other skill-based performance areas is an important step to address expanding accreditation standards throughout the health professions mandating interprofessional education for pre-licensure health professional students.

## Introduction

Interprofessional practice and education (IPE) has been identified as a mechanism capable of impacting the Triple Aim in healthcare; namely, improving the patient experience with care, improving the health of populations, and reducing the per capita cost of health care (Berwick et al., 2008). The IPE movement in the United States is well underway, bolstered by decades of work from the National Academy of Medicine (formerly the Institute of Medicine; IOM, 1972, 2001a,b, 2003), professional academic associations [Interprofessional Education Collaborative (IPEC, 2011a)], and federal legislation [House Office of the Legislative Counsel (HOLC, 2010)] that gave rise to the National Center for IPE based in Minneapolis, Minnesota [National Center for Interprofessional Practice and Education (NCIPE, 2016)]. Accrediting bodies across the health professions have also joined the IPE movement [Zorek and Raehl, 2013; Health Professions Accreditors Collaborative (HPAC, 2014)], mandating that academic institutions prepare their students for team-based care. Accreditation standards for pharmacy education, for example, require IPE across the continuum of the curriculum, from early didactic educational experiences through terminal experiential (i.e., clinical) ones [Accreditation Council for Pharmacy Education (ACPE., 2016)]. Interprofessional experiential education (IEE) holds particular promise to advance IPE, given its proximity to what the National Center refers to as the “Nexus,” where education and practice intersect [National Center for Interprofessional Practice and Education (NCIPE, 2016)].

Anderson and colleagues have argued that despite labor and cost barriers, the primary focus of IPE in the health professions should be within the experiential education setting (Anderson and Thorpe, 2010). We subscribe to this argument for several reasons. First, experiential learning allows students to be fully immersed in their future professional environment and learn aspects of their job beyond that which can be learned passively in classroom settings (Anderson and Thorpe, 2010). Students in this environment can experience interprofessional teamwork first hand, in the context of direct patient care. This may allow students to develop and hone skills specific to their career path. Moreover, as the healthcare system in the United States continues to evolve, the need for interprofessional collaboration will only increase [Interprofessional Education Collaborative (IPEC, 2011b)]. Shortages of resources, an aging population with multiple chronic conditions, as well as new scientific discoveries require both providers and non-clinical members of the healthcare team to work together in a cooperative manner [Interprofessional Education Collaborative (IPEC, 2011a; Nandan and Scott, 2014)]

As expectations for interprofessional practice increase, so too does the importance of new educational practices to prepare pre-licensure health professional students for collaborative practice upon entry into the workforce. A deeper exploration of potential best practices highlights the need for theoretical frameworks upon which to build novel interprofessional education interventions. Since consensus exists, in the form of the Interprofessional Education Collaborative's (IPEC's) core competencies expert panel report, on the skills required for interprofessional collaborative practice, we sought to identify a skill-based theoretical framework that might serve as a foundation to foster advancement of interprofessional experiential education (IEE). The theory of deliberate practice surfaced quickly in our cursory search, as it has been applied to the development of expertise in a wide array of skill-based disciplines, including chess, music, sports, and healthcare (Krampe and Ericsson, 1996; Moulaert et al., 2004; Ericsson, 2005, 2007a). The primary aim of this project, thus, was to integrate synergistic deliberate practice principles and IPEC competencies into a framework upon which educational models within IEE might be built.

## Methodology

A literature search of CINAHL, ERIC, and MEDLINE databases was completed using the keywords “deliberate practice” and “interprofessional education,” both individually and in combination. Articles that discussed deliberate practice or interprofessional education were cataloged for review. Relevant articles were selected from the catalog based on support for the premise of the project; that is, the integration of deliberate practice and interprofessional education principles into a framework to support development of IEE. Defining characteristics of deliberate practice were distilled with particular emphasis on their application to the Interprofessional Education Collaborative's (IPEC) core competencies. Recommendations for IEE development were identified through the synthesis of deliberate practice principles and IPEC competencies.

## Results

The findings guiding our proposed framework were derived from a total of 37 articles and reports with 27 pertaining to deliberate practice and 10 discussing IPE. The key elements of deliberate practice identified across these 27 articles are best described in narrative form. The theory of deliberate practice emphasizes, in the pursuit of expertise, quality over quantity of experiences and values the student's holistic ability to process, and integrate improvements in targeted skills (Ericsson et al., 1993). While the initial concept originated long ago, psychologist K. Anders Ericsson has published pioneering work in recent decades establishing the components of deliberate practice and the characteristics it entails (Ericsson and Charness, 1994; Ericsson, 1998, 2007b, 2008, 2015; Ericsson et al., 2009).

Fundamentally, the theory of deliberate practice posits that development of expertise requires incorporating a self-reflective feedback loop into the skill delivery or development (i.e., practice) process, rather than simply performing a task repetitively until mastered. To achieve maximal efficiency, time for self-reflection, and instantaneous feedback are vital for allowing the learner to self-adjust and make improvements before engaging in the next task. Mastery is thus achieved through repeated cycles of focused practice and self-editing, with each cycle emphasizing one or more aspects of a desired skill.

**Figure 1:**
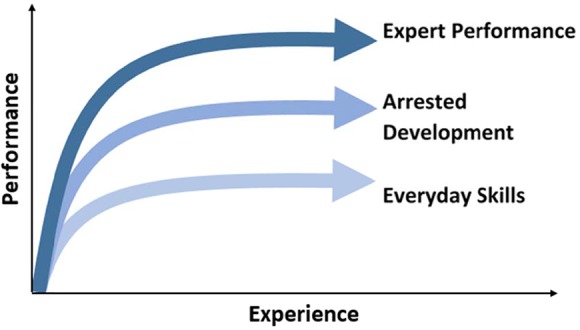
**Deliberate Practice and Performance**. Visual representation of processes leading to expert performance, arrested development, and automaticity involved in everyday skills. Adapted from Ericsson (2008).

With sufficient investment of time and dedication to the principles of deliberate practice, individuals can develop expertise in targeted areas (Figure 1). However, if the individual abandons these principles during the course of their career, arrested development can cause mastery to languish (Ericsson, 1998). This self-aware method of learning and evaluation is applied as a lifelong learning process toward developing expertise (Krampe and Ericsson, 1996). One important requirement for successful implementation of deliberate practice is a qualified teacher or preceptor to guide the process, shaping the student's reflections, and providing feedback. Preceptorship of students on clinical rotations in the latter stages of degree programs is a hallmark of health professions education. Thus, at least conceptually, the educational infrastructure exists for the integration of deliberate practice principles into IEE.

The IPEC competency framework includes 38 competencies dispersed across the following four domains: values/ethics for interprofessional practice (10 competencies), roles/responsibilities (9 competencies), interprofessional communication (8 competencies), and teams and teamwork (11 competencies) [Interprofessional Education Collaborative (IPEC, 2011a)]. Synthesis of deliberate practice principles and these competencies yielded seven areas of overlap, with at least one overlapping area from each IPEC domain. All of these, importantly, are conducive to learning opportunities present within the experiential education setting:

- Values/Ethics
  ◦ *Competency 7:* Demonstrate high standards of ethical conduct and quality of care in one's contributions to team-based care.

- Roles/Responsibilities
  ◦ *Competency 5:* Use the full scope of knowledge, skills, and abilities of available health professionals and healthcare workers to provide care that is safe, timely, efficient, effective, and equitable.
  ◦ *Competency 8:* Engage in continuous professional and interprofessional development to enhance team performance.

- Interprofessional Communication
  ◦ *Competency 4*: Listen actively, and encourage ideas and opinions of other team members.
  ◦ *Competency 7:* Recognize how one's own uniqueness, including experience level, expertise, culture, power, and hierarchy within the healthcare team, contributes to effective communication, conflict resolution, and positive interprofessional working relationships.

- Teams and Teamwork
  ◦ *Competency 8:* Reflect on individual and team performance for individual, as well as team, performance improvement.
  ◦ *Competency 11:* Perform effectively on teams and in different team roles in a variety of settings.

**Figure 2:**
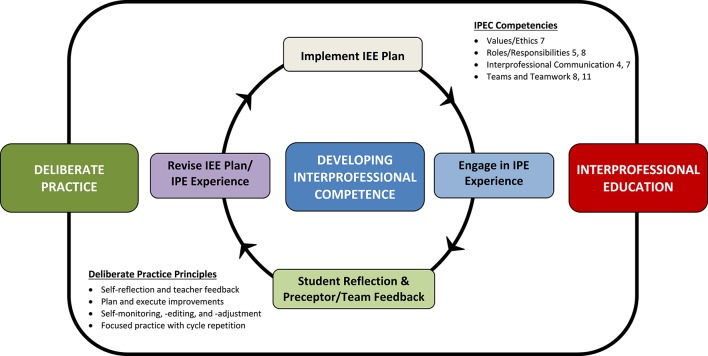
**Overview of Process Integrating Deliberate Practice and Interprofessional Experiential Education**. Developing interprofessional competence on clinical rotations begins with an individualized interprofessional experiential education (IEE) plan that is tailored to each student's unique strengths and weaknesses, followed by active participation in an interprofessional education (IPE) experience. Post-experience self-reflection is completed and preceptor/team feedback is obtained. Using this information, the student and preceptor collaborate to revise the IEE plan, and IPE experience before repeating the cycle again. This cycle is grounded in principles of deliberate practice and interprofessional education and targets Interprofessional Education Collaborative (IPEC) competencies. The specific text from each targeted IPEC competency follows: **Values/Ethics 7**, Demonstrate high standards of ethical conduct and quality of care in one's contributions to team-based care; **Roles/Responsibilities 5**, Use the full scope of knowledge, skills, and abilities of available health professionals and healthcare workers to provide care that is safe, timely, efficient, effective, and equitable; **Roles/Responsibilities 8**, Engage in continuous professional and interprofessional development to enhance team performance; **Interprofessional Communication 4**, Listen actively, and encourage ideas and opinions of other team members; **Interprofessional Communication 7**, Recognize how one's own uniqueness, including experience level, expertise, culture, power, and hierarchy within the healthcare team, contributes to effective communication, conflict resolution, and positive interprofessional working relationships; **Teams and Teamwork 8**, Reflect on individual and team performance for individual, as well as team, performance improvement; **Teams and Teamwork 11**, Perform effectively on teams and in different team roles in a variety of settings.

**Figure 3:**
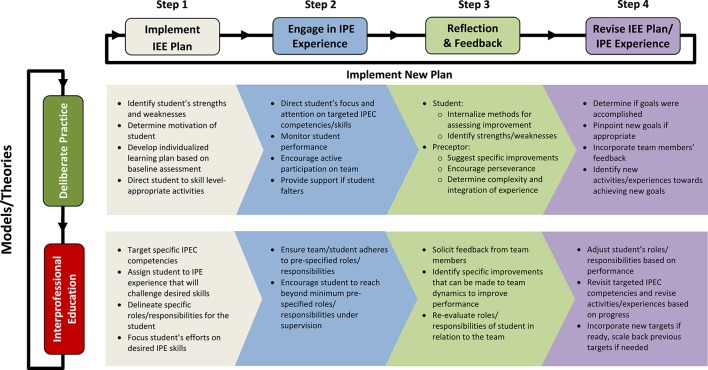
**Cyclical Stepwise Approach with Model/Theory-Specific Activities**. This figure is designed to begin in the Step 1 column. Here, the preceptor and student create and then implement the student's individualized interprofessional experiential education (IEE) plan based on a baseline assessment of the student at the beginning of the clinical rotation, including which Interprofessional Education Collaborative (IPEC) competencies he/she would like to target. Guidance for each step is presented in bullet form, with attribution of recommendations identified according to model/theory according to rows. The stepwise approach continues until Step 4, at which point the IEE plan and IPE experience are evaluated and revised according to student progress. Once Step 4 is complete, the cycle begins anew with Step 1. This process is followed throughout the clinical rotation.

Having identified synergistic competencies, the next step involved creating a process through which the student might begin developing competence in these areas during a clinical rotation. We propose a cyclical four-step process to achieve this aim: (1) implement an IEE plan guided by the student's strengths/weaknesses and in consideration of the collaborative practice skills they wish to develop, (2) engage in IPE experiences that will challenge targeted skills according to the IEE plan, (3) embed frequent opportunities for student reflection and preceptor/team feedback within IEE plan, and (4) revise the IEE plan and the IPE experience based on insights gained during step 3. A visual overview of this process is provided in Figure 2, and a detailed stepwise schematic is provided in Figure 3.

## Discussion

The four-step process identified through this literature review and synthesis may be used to guide the development of new models of interprofessional experiential education. The purposeful development of new IEE models rooted in an established theory that has already been applied to other skill-based performance areas is an important step to address looming accreditation mandates throughout the health professions.

We believe that robust, meaningful IEE experiences require an intentional effort to design the educational experience in accordance with students' strengths/weaknesses and their specific learning goals. It is not enough to simply place a student on a team and expect them to develop competence in interprofessional practice. Principles of deliberate practice possess a high degree of synergy with IPE and, as we have attempted to demonstrate with this project, can be integrated into the interprofessional clinical learning environment as an educational framework upon which meaningful, intentional IEE experiences can be built. Our hope is that this framework will help clinical educators tailor their clinical rotations to best train students in interprofessional collaborative practice. The educational model proposed offers a mechanism through which preceptors might help their students begin to develop competence in this area through a proactive process; not only to observe the tasks of practitioners, but also to gain direct, customized experience to develop and continually refine critical skills that will help prepare them for team-based practice upon licensure. This model provides a pathway for activities to be tailored to fit each student's individual needs in order to maximize their professional development.

Educators in medicine and nursing have already used deliberate practice to teach and train their students (Clapper and Kardong-Edgren, 2012; Durning et al., 2013; Gauthier et al., 2015; Modi et al., 2015), and studies show a variety of direct and tangential benefits. These include improved expertise in taking care of critically ill patients (Whyte and Cormier, 2014), neonatal resuscitation (Cordero et al., 2013; Hunt et al., 2014), laparoscopic surgical competence (Crochet et al., 2011), surgical residency training (Bhatti and Ahmed, 2015), and improving communications during shift changes (Sawatsky et al., 2013). By incorporating all four aspects of deliberate practice into the training of future doctors and nurses, randomized controlled trials, and observational studies demonstrate that students develop expertise more rapidly. We believe that deliberate practice can hasten the acquisition of interprofessional competence, and that our framework can guide clinical educators on the creation and/or improvement of their interprofessional clinical rotations.

## Conclusion

Quality patient care is increasingly dependent on collaborative teamwork. While general consensus and accreditation guidelines underscore the importance of interprofessional education, to our knowledge, no specific theoretical frameworks have been proposed to facilitate students' acquisition of the core competencies for collaborative practice outlined by the Interprofessional Education Collaborative. Motivated by the growing trend of integrating interprofessional education into health professional education, we attempted through this project to develop such a framework, which we believe clinical educators can use to help their students begin the process of developing interprofessional expertise. The model proposed herein integrates principles of deliberate practice with synergistic interprofessional practice competencies suitable for skill development in an experiential education setting. We encourage clinical educators preparing their students for interprofessional collaborative practice to utilize this framework and to study/disseminate educational outcomes from its use.

## Author contributions

JZ conceived, designed, and directed this study, and JW completed the literature review. Both authors analyzed and synthesized results. The proposed model and manuscript were developed collaboratively.

### Conflict of interest statement

The authors declare that the research was conducted in the absence of any commercial or financial relationships that could be construed as a potential conflict of interest.
